# Characterization of Naturally-Occurring Humoral Immunity to AAV in Sheep

**DOI:** 10.1371/journal.pone.0075142

**Published:** 2013-09-24

**Authors:** Joseph Tellez, Kim Van Vliet, Yu-Shan Tseng, Jonathan D. Finn, Nick Tschernia, Graça Almeida-Porada, Valder R. Arruda, Mavis Agbandje-McKenna, Christopher D. Porada

**Affiliations:** 1 Department of Animal Biotechnology, University of Nevada, Reno, Nevada, United States of America; 2 Department of Biochemistry and Molecular Biology, University of Florida, Gainesville, Florida, United States of America; 3 University of Pennsylvania School of Medicine, the Children’s Hospital of Philadelphia, Philadelphia, Pennsylvania, United States of America; 4 Wake Forest Institute for Regenerative Medicine, Winston-Salem, North Carolina, United States of America; University of Kansas Medical Center, United States of America

## Abstract

AAV vectors have shown great promise for clinical gene therapy (GT), but pre-existing human immunity against the AAV capsid often limits transduction. Thus, testing promising AAV-based GT approaches in an animal model with similar pre-existing immunity could better predict clinical outcome. Sheep have long been used for basic biological and preclinical studies. Moreover, we have re-established a line of sheep with severe hemophilia A (HA). Given the impetus to use AAV-based GT to treat hemophilia, we characterized the pre-existing ovine humoral immunity to AAV. ELISA revealed naturally-occurring antibodies to AAV1, AAV2, AAV5, AAV6, AAV8, and AAV9. For AAV2, AAV8, and AAV9 these inhibit transduction in a luciferase-based neutralization assay. Epitope mapping identified peptides that were common to the capsids of all AAV serotypes tested (AAV2, AAV5, AAV8 and AAV9), with each animal harboring antibodies to unique and common capsid epitopes. Mapping using X-ray crystallographic AAV capsid structures demonstrated that these antibodies recognized both surface epitopes and epitopes located within regions of the capsid that are internal or buried in the capsid structure. These results suggest that sheep harbor endogenous AAV, which induces immunity to both intact capsid and to capsid epitopes presented following proteolysis during the course of infection. In conclusion, their clinically relevant physiology and the presence of naturally-occurring antibodies to multiple AAV serotypes collectively make sheep a unique model in which to study GT for HA, and other diseases, and develop strategies to circumvent the clinically important barrier of pre-existing AAV immunity.

## Introduction

Adeno-associated viruses (AAVs) have attracted considerable interest in the field of gene therapy because they possess many characteristics that make them excellent vectors for gene transfer. Their genome is easily manipulated, AAV particles can be purified at very high titers, and they can then be lyophilized for easy handling/storage [1-3]. AAV efficiently transduces both proliferating and quiescent cells, and numerous serotypes exist in nature with differing tropisms, permitting some degree of tissue-targeting [4-7]. Also, the general consensus has been that AAVs may be inherently safer than many other viral-based vectors, since they are non-pathogenic and possess relatively low innate immunogenicity. Because genomic integration of AAV vectors is rare [8-11], the risk of insertional mutagenesis with AAV vectors is greatly reduced compared to retroviruses. These collective features have enabled AAV vectors to effectively correct a wide range of diseases in animal models, which, in turn, has prompted numerous clinical trials, in the hopes of safely achieving long-term expression of a variety of therapeutic proteins in human patients. An ongoing clinical gene therapy trial for hemophilia B [12,13] clearly highlights the tremendous potential of AAV-based vectors for the treatment of human disease.

However, many of the serotypes of AAV commonly employed in gene therapy procedures ubiquitously infect humans, generating pre-existing immunity against the AAV capsid proteins that precludes efficient transduction following intravascular administration, and/or induces CTL responses to the transduced target tissue [14-21]. While newborn dogs have been reported to exhibit pronounced selective immunity to AAV6 [22], most animals, with the exception of some non-human primates [19,23] and, perhaps the pig [24], do not appear to harbor very robust pre-existing immunity/endogenous antibodies to many of the serotypes of AAV commonly employed as gene delivery vectors. This lack of pre-existing immunity to AAV could explain, in part, why many highly successful studies conducted in a variety of animal models have not translated into clinical success when similar approaches have been applied to human patients [25,26]. Sheep have been used for decades as a model to study a broad range of disease states, and a high degree of clinical predictability has consistently been observed. Recently, sheep were utilized as a large animal model for myocardial gene delivery using molecular cardiac surgery with recirculating delivery (MCARD) [27], as well as for testing rAAV2/1-SERCA2a vectors, in an experimental heart failure model [28]. Novel in utero gene therapy studies aimed at correcting congenital diseases that develop perinatally recently also utilized sheep as the large animal model [29]. Moreover, we have re-established a line of sheep with severe hemophilia A with a null mutation in the FVIII gene [30] to provide a suitable large animal model for testing novel rAAV vectors for this condition. A recent publication provided evidence that commercially available sheep sera may harbor very low levels of antibodies to a limited number of AAV serotypes [24], suggesting at least a subset of sheep may harbor immunity to AAV. Here we report studies performed to address whether sheep harbor naturally-occurring antibodies to the same serotypes of AAV as healthy humans [20,21], and to characterize the nature of this pre-existing immunity. Enzyme-linked immunosorbent assays (ELISA) demonstrated the existence of naturally occurring antibodies against several AAV serotypes, AAV1, AAV2, AAV8, and AAV9. In addition, sera from three of the sheep screened neutralized transduction by AAV2, AAV8, and AAV9 to different levels, with AAV2 being the most affected. These observations recapitulate reports for the human response against the AAVs, with antibodies against AAV2 being the most prevalent in the human population [20]. Finally, peptide array based epitope mapping identified common AAV serotype antigenic epitopes that were recognized by different sheep sera as well as those that were unique to each sheep. Similar to the human immune response against the AAVs, the sheep reactivity against the different AAV serotypes was varied. To our knowledge, this is the first study to delineate, in a large animal (sheep) model, the epitopes within the AAV capsid that are responsible for triggering naturally-occurring functional antibodies to multiple serotypes of AAV commonly employed as gene therapy vectors. The close parallels between human and sheep physiology and the presence of these antibodies, suggest that sheep may represent an ideal large animal model in which to study gene therapy in the context of pre-existing immunity to AAV, and to develop novel strategies for circumventing this clinically important immunologic barrier.

## Materials and Methods

### Ethics Statement

This study was approved by the University of Nevada, Reno Institutional Animal Care and Use Committee.

### Indirect enzyme-linked immunosorbent assay (ELISA) detection of anti-AAV-antibodies in sheep serum

Serum collected from the peripheral blood of six healthy adult female Merino-Rambouillet sheep bred and raised at the University of Nevada Agricultural Experiment Station was used as the primary antibody in an indirect ELISA. Sheep IgG (Rockland, Gilbertsville, PA, USA) was used to prepare a standard curve (31.25-4000ng/ml) and AAV1-FIX, AAV2-FIX, AAV5-GFP, AAV6-GFP, AAV8-Ova and AAV9-Ova (AAV5 and 6 were purchased from Vector Biolabs, Philadelphia, PA, USA; AAV1, 2, 8, and 9 were kindly provided by Dr. Roland Herzog) served as the antigen (2.5x10^9^ vector genomes/well). Both the antibody and virus samples were fixed to the appropriate wells of a 96-well plate with 50µl Coating Solution (KPL, Gaithersburg, MD, USA), the plate was sealed, and then incubated overnight at 4°C. The plate was washed 3 times with phosphate-buffered saline (PBS: 137mM NaCl, 2.7mM KCl, 1.5mM KH_2_PO_4_, 8mM NaH_2_PO_4_) containing 0.05% Tween-20, and blocked with blocking/dilution buffer (PBS, 6% BSA, 0.05% Tween-20) for 1hr at RT. The plate was again washed three times with PBS containing 0.05% Tween-20. Sheep serum was diluted 1:20 in blocking/dilution buffer, and 50µl was added to each of the experimental wells. Wells containing no viral particles served as controls for non-specific binding of sheep serum and secondary antibody to the microplate wells. The plate was incubated for 2hr at 37°C and then washed three times in PBS containing 0.05% Tween-20. Rabbit-anti-sheep IgG conjugated to horse radish peroxidase (HRP) secondary antibody (Southern Biotech, Birmingham, AL, USA) was diluted 1:500 in blocking/dilution buffer and added to the wells to detect bound antibodies in the sheep sera. The plate was incubated again for 2hr at 37°C and washed three times in PBS containing 0.05% Tween-20. One hundred microliters of SIGMA*FAST*™ OPD substrate (o-phenylenediaminedihydrochloride, Sigma-Aldrich, St. Louis, MO, USA) was added to each well, and the plate was incubated for 1 hr at 37°C, with OD readings taken every 15 minutes in a 3550-UV Microplate Reader (Bio-Rad, Hercules, CA, USA) until the standard curve was linear in a semi-log scale.

### Luminescence-based AAV neutralization assay

HEK-293 cells were seeded at 20,000 cells/well in 200µl of Dulbecco’s Modified Eagle Medium (DMEM; Invitrogen/Life Technologies, Grand Island, NY, USA) containing 10% fetal bovine serum (FBS; HyClone/Thermo Scientific, Logan, UT, USA), 1% glutamine (Invitrogen/Life Technologies), and 1% penicillin/streptomycin (Invitrogen/Life Technologies) in 96-well plates (E&K Scientific Products, Inc., Santa Clara, CA, USA). Cells were then incubated overnight at 37°C, 5% CO_2_.

On the following day, sera from the 3 sheep exhibiting different titers of antibodies (lowest, medium, and highest) to the various AAV serotypes were thawed on ice and then heat-inactivated for 30 minutes at 56°C. A series of dilutions (from 1:3.1 to 1:3160) of eah serum sample was then prepared in heat-inactivated FBS, with pooled normal human plasma diluted in the same fashion used as a positive control/standard. AAV2, AAV8, and AAV9 vectors encoding the Renilla luciferase gene under the control of the cytomegalovirus/chicken beta-actin hybrid (*CBA*) promoter were used. Eighteen microliters of each diluted AAV vector (8e7 vg for AAV2, 1.5e9 vg for AAV 8 and 9) was mixed with 18µl of each of the diluted serum samples, and incubated for 1hr at 37°C. An aliquot (7.5µl) of each of the serum/AAV vector mixtures was then transferred to an individual well of the tissue culture plate containing the HEK-293 cells, plating each sample dilution in triplicate (AAV2 MOI = ~800, AAV8/9 MOI = ~15,000). The plate was incubated overnight (16-24hr) at 37°C, 5% CO_2_.

The next morning, 5x Renilla luciferase lysis buffer (Promega Corp., Madison, WI, USA) was thawed, diluted to a 1x working solution with diH _2_O, and kept on ice until use. The medium was removed from the HEK-293 cell plates, and each well was gently washed with 200µl of 1x PBS. Forty microliters of 1x lysis buffer was added to each well, and the plates were then agitated for 15 minutes at room temperature on a Clay Adams *Nutator* Mixer (BD Diagnostics, Franklin Lakes, NJ, USA). The Renilla luciferase assay buffer was thawed and used to prepare a 1x solution of Renilla luciferase assay substrate (both from Promega). Following priming of the luminometer (GloMax®-96 Microplate Luminometer, Promega Corp.) with substrate buffer, the plates were inserted and read with an integration time of 2 seconds, an injection volume of 50µl, an injection rate of 333µl/second, and a delay between injection and measurement of 1 second. The data generated was automatically saved within a Microsoft Excel spreadsheet.

### Peptide library generation and peptide epitope-mapping

ProArray™ peptide microarrays containing 557 15mer peptides overlapping by 10 amino acids and covering the entire VP1 sequence from the capsids of AAV2, AAV5, AAV8, and AAV9 (ProImmune, Inc., Oxford, UK) were used to screen reactivity of individual sheep sera. All ProArrays™ were blocked for 2hr using validated, proprietary blocking buffer, and were then incubated with the individual sheep sera (collected from the 3 sheep used for the Luminescence-Based AAV neutralization assay detailed above) diluted in blocking buffer (1:200 dilution, total assay volume 400µL), with a fluorescently labeled secondary antibody, which binds selectively to an antigen-specific antibody (i.e. anti-sheep) (control sample) or with blocking buffer only (control buffer). The ProArrays™ were washed five times with blocking buffer and five times with PBS, followed by incubation with Cy3-labeled AffiniPure Rabbit Anti-Sheep-IgG (H+L) secondary antibody (Stratech Scientific, Ltd., Newmarket Suffolk, England) at a concentration of 1µg/ml. Following incubation with the secondary antibody, five washing steps with blocking buffer were performed, followed by five washing steps with PBS, and five washing steps with analytical grade water. The ProArrays™ were then thoroughly dried and scanned using a high-resolution fluorescence scanner (Innoscan 700; Innopsys, Carbonne, France). Laser settings and applied resolution were identical for all measurements performed. The resulting images were processed and analyzed using the integrated Mapix spot-recognition software (Arrayit Corporation (ARYC), Sunnyvale, CA), showing the signal intensity (range between 0 and 65536 Light Units) as single measurements for each peptide. Each spot-feature was analyzed for signal intensities for pixels within recognized spots and for the pixels surrounding the recognized spots (background). The median of signal intensities of the background was subtracted from the median of signal intensities for pixels within recognized spots, resulting in corrected median values (signal minus background). Mean values of the corrected median of signal intensities from 3 identical subarrays were used for the data analysis. The signals from the control sample (anti-sheep secondary antibody) were subtracted from the sheep sera signals and peptides with intensity values greater than 7,500 LU were considered positive binders. The VP and capsid regions of the peptides identified by the peptide microarray screening were visualized in the structures of AAV2, AAV5, AAV8, and AAV9 capsids, determined by X-ray crystallography (PDB identifiers 1LP3, 3NTT, 2QA0, and 3UX1, respectively), using the Coot program [31] and images to show their locations were generated using the PyMol program (The PyMOL Molecular Graphics System, Version 1.3, Schrödinger, LLC.).

## Results

### Detection of naturally-occurring antibodies to AAV in sheep

**Figure 1:**
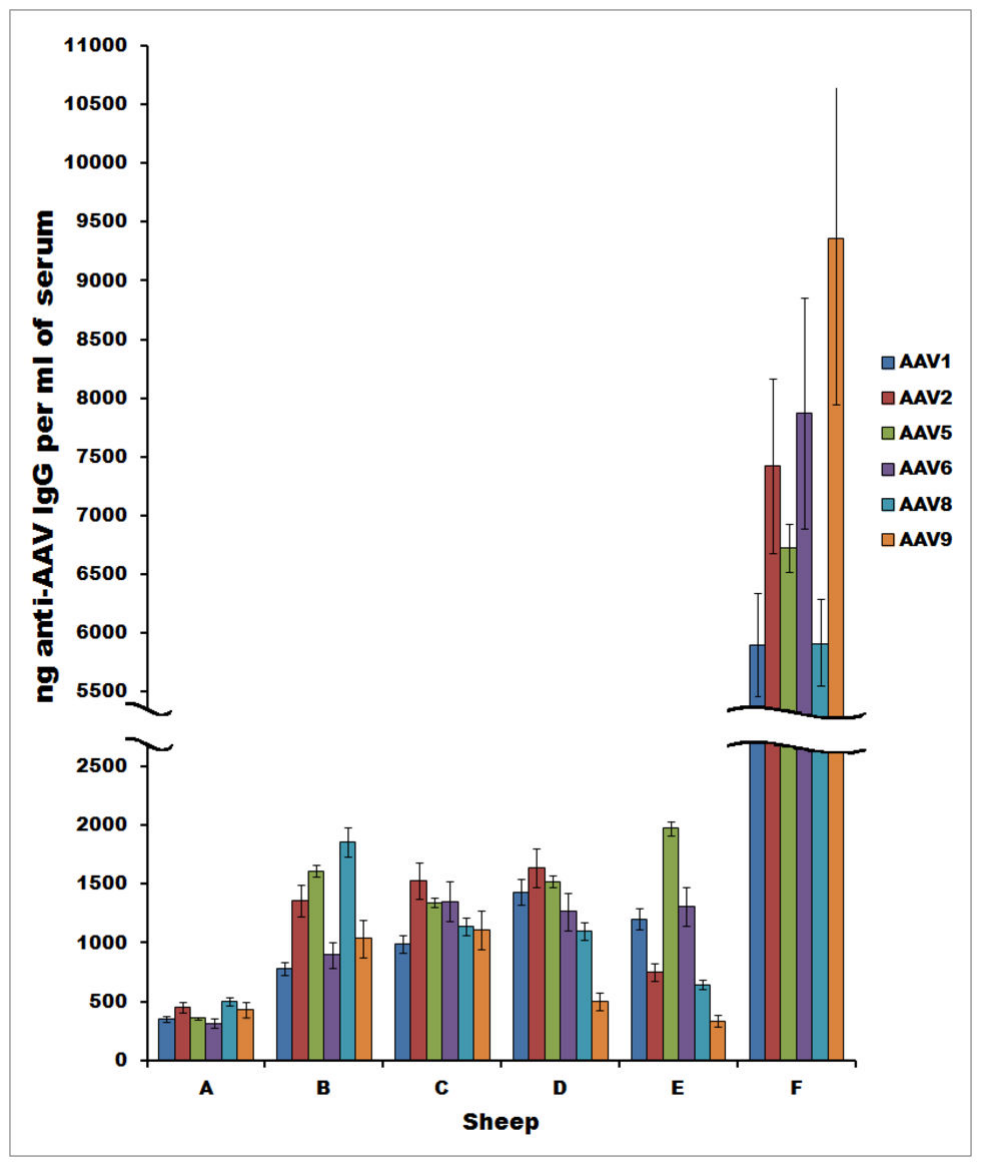
Presence in sheep of antibodies that recognize common AAV serotypes. ELISAs were performed on sera from a panel of 6 healthy sheep using AAV1, AAV2, AAV5, AAV6, AAV8, or AAV9 particles as the antigen. Results are presented as concentration of IgG against each AAV serotype in each individual animal’s serum. All samples were run in triplicate, and the depicted values represent the mean of 3 separate experiments.

To assess whether sheep harbor naturally-occurring antibodies against the serotypes of AAV commonly employed as gene therapy vectors, ELISAs were performed on sera from a panel of 6 healthy Merino-Rambouillet sheep using AAV1, AAV2, AAV5, AAV6, AAV8, and AAV9 particles as the antigen. As shown in Figure 1, these analyses revealed that healthy sheep harbored naturally-occurring antibodies to all 6 AAV serotypes tested. The titers against the different serotypes varied greatly from sheep-to-sheep, similar to the observation of human sera reactivities to AAVs. Specifically, one sheep (Sheep F) exhibited a very high level (>5900ng/ml) of IgG against all 6 AAV serotypes tested. In contrast, levels of anti-AAV IgG in the remaining 5 sheep were varied, with Sheep C exhibiting moderate/low (992-1530ng/ml) IgG levels against all 6 AAV serotypes, Sheep D moderate/low (500-1637ng/ml) IgG levels against all serotypes except AAV9, Sheep B and E moderate/low (335-1972ng/ml) IgG levels against only 3-4 of the tested serotypes, and sheep A only low (<500ng/ml) IgG levels against all 6 AAV serotypes.

### Anti-AAV antibodies in sheep neutralize transduction

**Figure 2:**
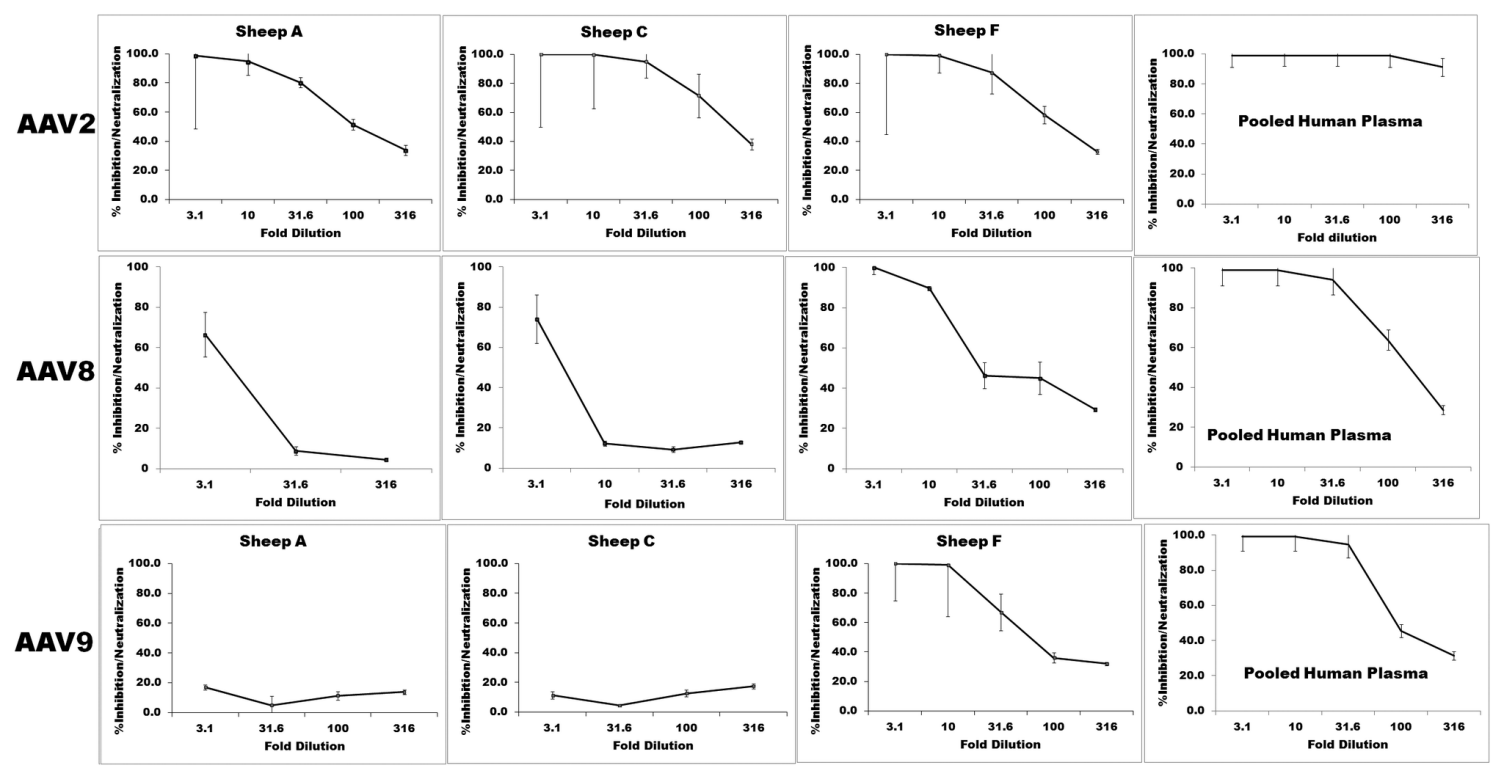
Neutralizing activity of anti-AAV antibodies. To assess the functional significance of the detected antibodies in the context of AAV-based gene delivery, a luciferase-based neutralization assay was performed on sera from sheep A, sheep C, and sheep F against AAV2, AAV8, and AAV9. Pooled normal human plasma diluted in the same fashion served as a positive control/standard. Data are presented as the titer (dilution) of the serum from each sheep versus the percent inhibition observed when a known quantity of luciferase-encoding AAV vector of each serotype was exposed to serum from each sheep and then used to transduce HEK-293 cells *in* *vitro*. The values presented are the mean obtained running all samples in triplicate + SEM.

To assess the functional significance of the anti-AAV antibodies in the sheep sera in the context of AAV-based gene delivery, a luciferase-based neutralization assay was performed (see Materials and Methods) on sera from three sheep exhibiting, low (sheep A), medium (sheep C), and high (sheep F) IgG titers against AAV2, AAV8, and AAV9. As shown in Figure 2, despite having widely disparate levels of IgG against AAV2 by ELISA, sera from all three of the animals tested were neutralizing (1:100 to 1:316 dilution) against AAV2, similar to the high neutralizing activity seen with pooled human plasma against this AAV serotype. In agreement with the IgG levels seen by ELISA (Figure 1), only the sera from Sheep F was neutralizing against AAV8 and AAV9 (1:31, and 1:100 dilution, respectively), while the sera from Sheep A and Sheep C were not neutralizing (1:1 to1:3 dilution) against these two AAV serotypes. While the pooled human plasma neutralized both AAV8 and AAV9, the neutralizing titers against these two serotypes were lower than those observed against AAV2.

### Identification of capsid epitopes recognized by anti-AAV IgG in sheep

**Figure 3:**
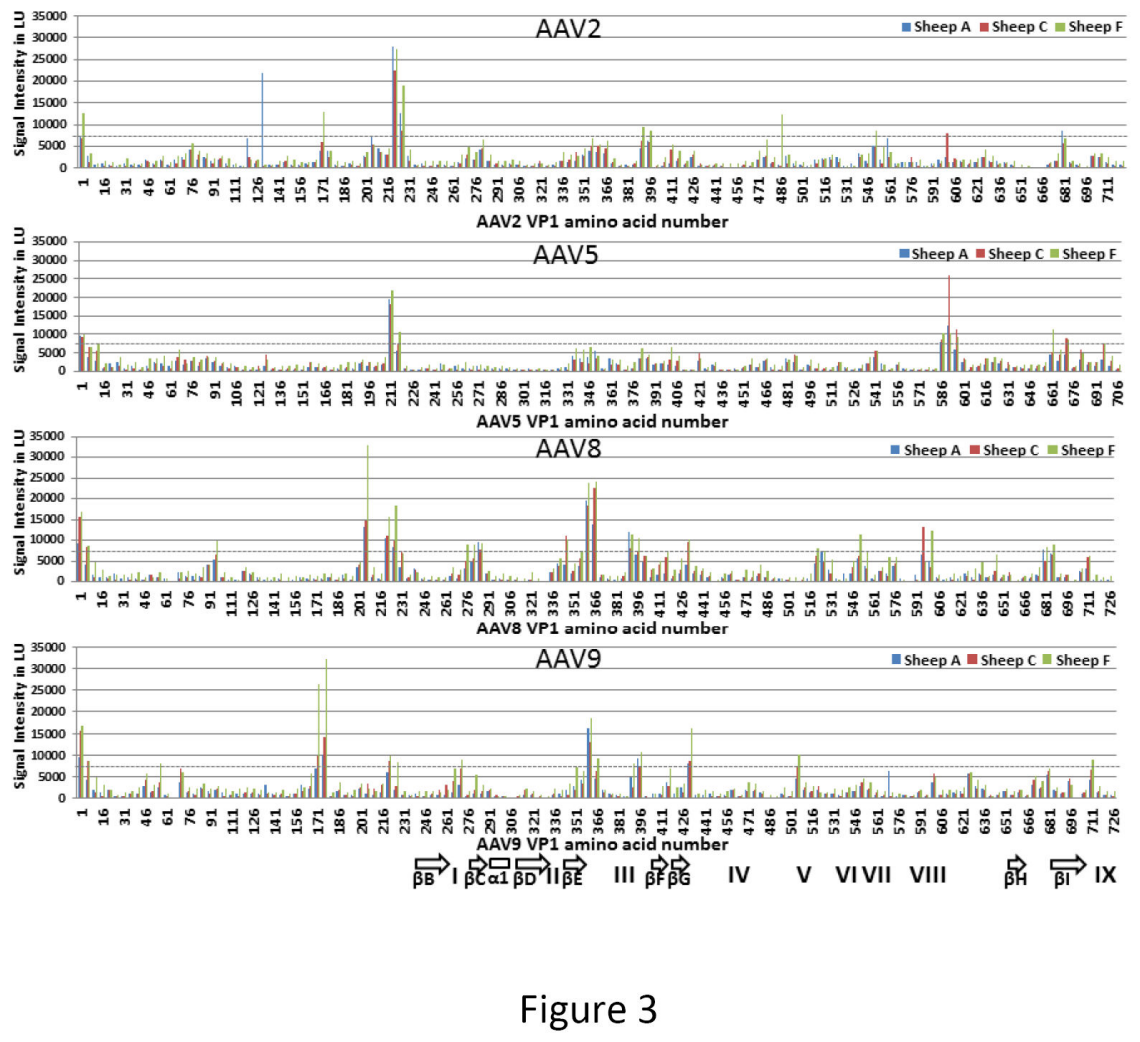
Identification of peptides responsible for antibody binding to AAV. To identify the epitopes within the AAV capsid recognized by the antibodies present within the sera of healthy sheep, a comprehensive peptide library covering the entire VP1 sequence from the capsids of AAV2, AAV5, AAV8, and AAV9 was probed with sera from the three sheep utilized for the neutralization assay (sheep A, sheep C, and sheep F). The histograms depicted in this figure show the results obtained when arrays containing peptides for AAV2, AAV5, AAV8, or AAV9 were probed with sera from sheep A, sheep C, or sheep F. The histogram was plotted based on the amino acid number of the first amino acid in the reactive peptide. VP1 numbering for each serotype was used.

**Figure 4:**
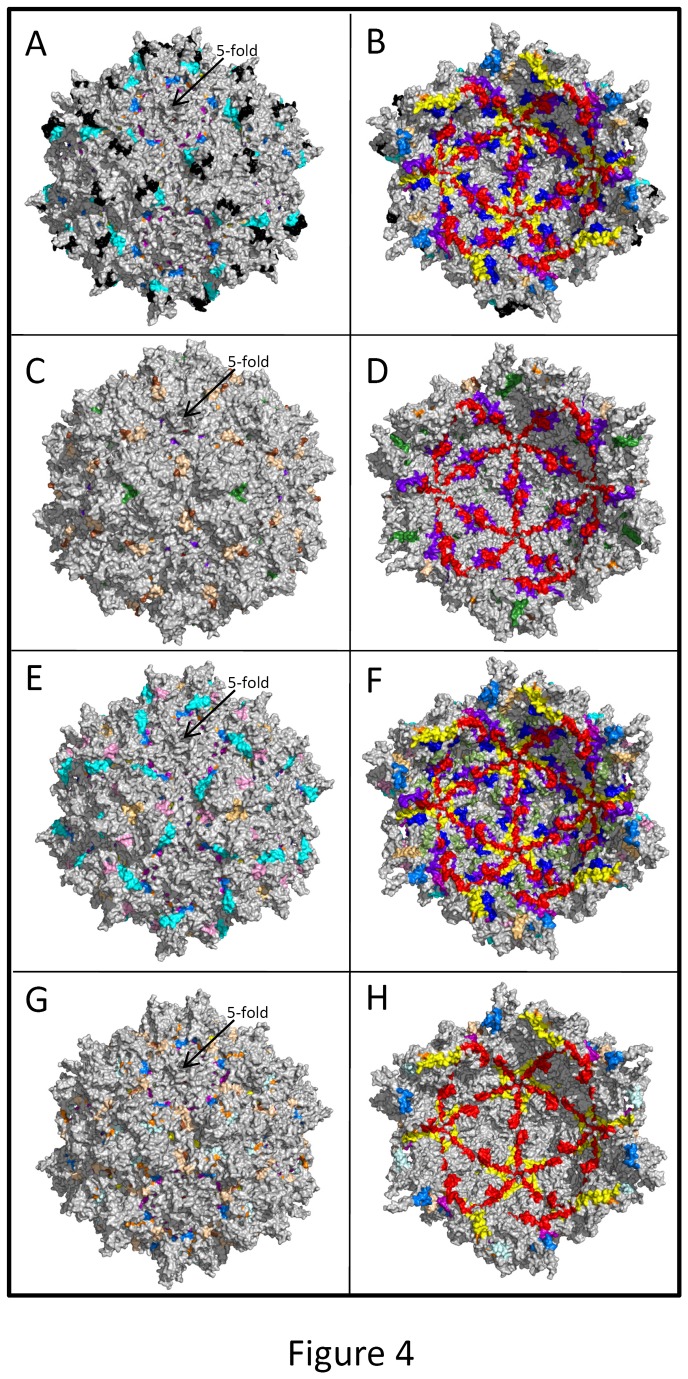
Locations of peptide epitopes on the AAV capsid. Surface density images are shown for the outside of AAV2, AAV5, AAV8, and AAV9, (A), (C), (E), and (G) respectively (left panels). Inside surface density images which are rotated 180° relative the outside image and viewed approximately along the icosahedral 2-fold axis for AAV2, AAV5, AAV8 and AAV9 in (B), (D), (F), (H) respectively (right panels). Epitopes common to AAV2, AAV5, AAV8, and AAV9 are shown in red (epitope b, 221-VGNSSGNWHCDSTWMGDRVI-240– Table 1), purple (epitope c, 363-PPFPADVFMIPQYGYL-378– Table 1), yellow (epitope d, 388-GRSSFYCLEYFPSQMLRTGNNFT-410– Table 1), dark green (epitope e, 586-NLQEIVPGSVWMERDVYLQGPIWAK-610 – Table 1) (RCSB PDB accession 1LP3). In A-C, the two epitopes common AAV2, AAV5, AAV8, and AAV9 are colored purple, Epitope b in Table 1: 221-VGNSSGNWHCDSTWMGDRVI-240; and blue, Peptide 337 in Table 1: 273-FGYSTPWGYFDFNRF-287. In D-F, the five epitopes common AAV2, AAV8, and AAV9 are colored purple, Peptide 338 in Table 1: 278-PWGYFDFNRFHCHFS-292; blue, Peptide 339 in Table 1: 283-DFNRFHCHFSPRDWQ-297; yellow, Epitope c in Table 1: 363-PPFPADVFMIPQYGYL-378; red, Epitope d in Table 1: 388-GRSSFYCLEYFPSQMLRTGNN-408; and black Peptides 368 and 499 in Table 1: 428-QSLDRLMNPLIDQYLY-443. The equivalent surface accessible peptide epitopes identified for the AAV2, AAV5, AAV8, and AAV9 are shown on an AAV2 capsid surface generated with 60 copies of VP3 (accession number as given in Figure 4). The epitopes are colored differently based on the AAV serotypes: blue for AAV2 (AAV2 residues 486-QRVSKTSADNNNSEY-500, peptide 97 in Table 1), black for AAV5 (AAV5 residues 586-NLQEIVPGSVW**MERD**-600, peptide 262 (epitope e) in Table 1), green and dark green for two epitopes on AAV8, (green: AAV8 residues 551-ARDNADYSDVMLTSE-565, peptide 392 in Table 1; dark green: AAV8 residues 596-GTVNSQGALPGMVWQ-610, peptide 401 in Table 1), and brown for AAV9 (AAV9 residues 506-ASSWALNGRNSLMNP-520, peptide 514 in Table 1).

Our results demonstrated the presence of antibodies recognizing multiple AAV serotypes, raising the question of whether this seemingly broad immunity to AAV was the result of antibodies against an epitope(s) common to all of the tested AAV serotypes, or whether sheep harbor antibodies that recognize multiple distinct epitopes that are unique to each serotype’s capsid. To address this question, we performed epitope mapping to define the peptides within the capsids of AAV2, AAV5, AAV8, and AAV9 recognized by these antibodies. As detailed in the Materials and Methods, a peptide library covering the entire VP1 sequence from the capsids of AAV2, AAV5, AAV8, and AAV9, was synthesized by ProImmune, Inc. (Oxford, UK) and probed with sera from the three sheep utilized for the neutralization assay (sheep A, sheep C, and sheep F). The screening resulted in the identification of numerous immunogenic epitopes within the capsids of these viruses as positive hits. As can be seen in Figure 3, the antibodies present within the serum from each sheep recognized multiple distinct epitopes within the capsid, with varying degrees of affinity, the majority of which were common amongst the three sheep tested. The sequence information for each of the most immunogenic peptide epitopes identified with these arrays are shown in Table 1, along with the serotypes of AAV containing the given epitope, and the epitope’s amino acid position within the respective VP1. Regions of the capsid most likely to contain antigenic regions are included in peptides 26, 44, 45, 323, 326, 327, 354, 401, 402, 450, 451, and 485. Although many of the recognized epitopes in Table 1 were common to the capsids of all AAV serotypes tested, each animal also harbored antibodies to epitopes that were unique to each specific capsid, with Sheep F having the most reactivity, consistent with the ELISA and neutralizing antibody data. Using the AAV capsid structures determined by X-ray crystallography for AAV2, AAV5, AAV8, and AAV9 [32-34] the positive hit peptides (Table 1) were mapped on the capsid VP to determine their structural locations (Table 1 and Figure 4). Surprisingly, most of the epitopes were located within regions of the VP that are internal or buried within the capsid in conserved secondary structure elements (Figures 4B, 4D, 4F and 4H). However, consistent with the recognition of the AAV capsids in the ELISA and neutralization assay results, a number of the peptides recognized were located on the capsid surface and in regions analogous to previously mapped neutralizing antigenic epitopes in AAV2 and in AAV8 [35-37] (Table 1, Figures 4A, 4C, 4E and 4G). These results suggest that sheep harbor endogenous AAV, which induces immunity to both intact capsids and capsid epitopes that are presented following proteolysis during the course of infection.

**Table 1:** Positive Binders.

**Sheep**	**ID**	**Epitope Sequence**		**AAV Serotype**	**Amino acid# (VP1 #’s**)	**Epitope/Location**
F	1	**a**	**MAAD**GYLPDWLEDTL	AAV2	1-15	
A, C, F	145		MSFVDHPPDWLEEVG	AAV5	1-15	
A, C, F	287	**a**	**MAAD**GYLPDWLEDNL	AAV8, AAV9	1-15, 1-15	
C, F	288		YLPDWLEDNLSEGIR	AAV8	6-20	
F	304		ADAEFQERLQEDTSF	AAV8	96-110	
A	26		LVEEPVKTAPGKKRP	AAV2	131-145	
F	34		LNFGQTGDADSVPDP	AAV2	171-185	A69/ND
C, F	450		LNFGQTGDTESVPDP	AAV9	207-225	
A, C, F	451		TGDTESVPDPQPIGE	AAV9	208-225	
A	41		GSGAPMADNNEGADG	AAV2	209-225	
A, C, F	323		AGGGAPMADNNEGAD	AAV8	210-225	
A, C, F	187	**b**	VGNASGD**WHCDS**TWM	AAV5	211-225	IP-IS
F	188	**b**	GD**WHCDS**TWMGDRVV	AAV5	216-230	IP-IS
A, C, F	44	**b**	VGNSSGN**WHCDS**TWM	AAV2	221-235	IP-IS
A, C, F	326	**b**	GVGSSSGN**WHCDS**TW	AAV8	221-235	IP-IS
F	460	**b**	VGSSSGN**WHCDS**QWL	AAV9	221-235	IP-IS
A, C, F	45	**b**	GN**WHCDS**TWMGDRVI	AAV2	226-240	IP-IS
A, C, F	327	**b**	SGN**WHCDS**TWLGDRV	AAV8	226-240	IP-IS
F	461	**b**	GN**WHCDS**QWLGDRVI	AAV9	226-240	IP-IS
F	467		DNAYFGYSTPWGYFD	AAV9	271-285	B
F	337		FGYSTPWGYFDFNRF	AAV2, AAV5, AAV8, AAV9	273-287,264-278,276-290,275-289	A20-1/B
F	338		PWGYFDFNRFHCHFS	AAV2, AAV8, AAV9	278-292,281 - 295,280 -294	B
A, C, F	339		DFNRFHCHFSPRDWQ	AAV2, AAV8, AAV9	283-297,286 - 300,285 -299	B
C, F	351		FTDSEYQLPYVLGSA	AAV2, AAV8	343-357,346 - 360	MK/IS/B
A, C, F	354	**c**	HQGCLPPF**PADVFM**I	AAV8	361-375	B
A, C, F	485	**c**	EGCLPPF**PADVFM**IP	AAV9	361-375	B
A, C, F	355	**c**	PPF**PADVFM**IPQYGY	AAV2, AAV8, AAV9	363 - 377,366 -380,365–379	A20-2/B
F	486	**c**	PF**PADVFM**IPQYGYL	AAV2, AAV8, AAV9	364 - 378,367 -381,366–380	A20-2/B
A, C, F	360	**d**	GRSSFY**CLEY**FPSQM	AAV2, AAV8, AAV9	388-402,391 - 405, 390–404	IP-IS
F	491	**d**	RSSFY**CLEY**FPSQML	AAV9	391-405	IP-IS
F	78	**d**	SFY**CLEY**FPSQMLRT	AAV2, AAV8, AAV9	391 - 405,394 -408,393–407	MK/IS
F	361	**d**	Y**CLEY**FPSQMLRTGN	AAV2, AAV8, AAV9	393 - 407,396 -410,395–409	MK/IS
A, F	492	**d**	**CLEY**FPSQMLRTGNN	AAV2, AAV8, AAV9	394 - 408,397 -411,396–410	MK/IS
F	79		**EY**FPSQMLRTGNNFT	AAV2	396-410	MK/IS
F	365		YTFEDVPFHSSYAHS	AAV8	416-430	IS
C, F	368		QSLDRLMNPLIDQYL	AAV2, AAV8, AAV9	428 - 442,431 -445,430–444	3F,IS
A, C, F	499		SLDRLMNPLIDQYLY	AAV2, AAV8, AAV9	429 - 443,432 -446,431–445	3F,IS
F	97		QRVSKTSADNNNSEY	AAV2	486-500	C37-2/VRV,3F,OS
F	514		ASSWALNGRNSLMNP	AAV9	506-520	3F,OS,PB
F	386		NPGIAMATHKDDEER	AAV8	521-535	VRVI, OS,PB
F	110		NVDIEKVMITDEEEI	AAV2	551-565	VRVII, OS
F	392		ARDNADYSDVMLTSE	AAV8	551-565	VRVII, OS
A, C, F	262	**e**	NLQEIVPGSVW**MERD**	AAV5	586-600	3F,OS,PB
A, C, F	263	**e**	VPGSVW**MERD**VYLQG	AAV5	591-605	3F,B
C,F	264	**e**	W**MERD**VYLQGPIWAK	AAV5	596-610	IS,B
C	401		GTVNSQGALPGMVWQ	AAV8	596-610	OS
C	120		LPGMVWQDRDVYLQG	AAV2	601-615	IS/B
F	402		QGALPGMVWQNRDVY	AAV8	601-615	IS
F	277		QYSTGQVTVEMEWEL	AAV5	661-675	IS
C, F	279		MEWELKKENSKRWNP	AAV5	671-685	IS
A	136		EIEWELQKENSKRWN	AAV2	681-695	IS
A, F	418		VSVEIEWELQKENSK	AAV8	681-695	IS
F	419		EWELQKENSKRWNPE	AAV8	686-700	IS
C, F	284		PQFVDFAPDSTGEYR	AAV5	696-710	B
F	555		VEFAVNTEGVYSEPR	AAV9	711-725	OS

Positive hits from the peptide array were mapped on the surface of the AAV capsid to identify immunogenic regions. Five sequences that appeared frequently were designated epitopes a (MAAD), b (WHCDS), c (PADVFM), d (CLEY), e (MERD). Known AAV2 Epitopes are listed, including A20 Epitopes, A20-1, 271-HYFGYSTPWG-280; a20-2, 369-VFMVPQYGYL-378; A20-4, 566-RTTNPVATEQ-575; AAV2 A1 Epitope, 123-KRVLEPLGL-131; A69 Epitope, 171-LNFGQTGDADSV-182; AAV2 C37-B Epitopes, 493-SADNNNSEYSWT-502 AND 601-LPGMVWQDRD-610. Epitopes previously identified in Moskalenko, et al [43] are listed as MK. The location of the peptide on the AAV VP capsid structure was determined and IP-IS is Intrapentameric interface-Inner Capsid Surface, IS is Inner Capsid Surface, B is Buried in the assembled capsid, OS is outer capsid surface, PB is Partially Buried and ND is Not Determined. The inner surface residues are primarily located at interfaces, for example at monomer interfaces that make up pentermers; VRIV, VRV, VRVII, and VRVIII are surface loops that vary among serotypes as defined in Govindasamy, et al. [57] variable loops in Peptide for which there is no structural information are shaded in grey.

## Discussion

### Sheep as an animal model for studying the immune response to AAV gene therapy vectors

Preclinical studies in both small and large animal models have demonstrated that AAV-mediated gene transfer is safe and has resulted in long-term gene expression and phenotypic correction of a wide range of disorders (e.g. [2,8,9,11,12,27-29,38-40]). However, the host immune response represents a formidable barrier to achieving therapeutic efficacy in the clinic, due largely to the presence of pre-existing immunity to AAV capsids in humans as a result of previous exposure to AAV. A recent publication [24] provided evidence that sera from a variety of animals commonly employed for biomedical research, including sheep, harbor neutralizing antibodies to some serotypes of AAV. This study thus identified animal species suited for studying the immunologic aspects of AAV-based gene delivery evaluating strategies to circumvent pre-existing AAV immunity. Sheep have long been used as a model to study normal development and physiology, as well as a broad range of disease states, and results obtained with this model have consistently yielded a high degree of clinical predictability in humans. In this study, the humoral immunity to AAV observed in normal healthy sheep, based on capsid ELISA, mimicked the marked individual-to-individual titer variability of antibodies against AAV capsid that has been observed in the human population [20]. The neutralization assay from the three selected sheep with variable IgG response further confirmed the similarity between the sheep and human pre-existing AAV capsid immunity response when tested against AAV2, AAV8, and AAV9. These analyses revealed that, like most humans, sheep harbor relatively high titer neutralizing antibodies (NAB) to AAV2, but varied titers against AAV8 and AAV9. Prior studies have shown that even low levels of neutralizing antibody (1:5 to 1:10) were sufficient to completely abrogate AAV-mediated transduction [38,41], and even non-neutralizing antibodies negatively impacted upon transduction in vivo [42], consistent with our observations. These observations establish sheep as the first experimental animal model with significant NAB and IgG to AAV2 and AAV8, and thus support the use of sheep for future studies of the immune response to AAV gene therapy vectors.

### Specificity and cross-reactivity is common to sheep and AAV serotypes

Peptide-based array epitope screening defined peptides within the capsids of AAV2, AAV5, AAV8, and AAV9 recognized by the three sheep sera tested. The reactivity profiles were very similar between the sheep (Figure 3) with sheep F, containing the highest AAV antibody titer against the different serotypes, showing strongest reactivity against all the serotypes. Sheep F harbored antibodies that recognized multiple distinct epitopes that are unique to each capsid serotype as well as those that were common to all of the tested AAV serotypes (Table 1). The low and medium AAV antibody titer sera (Sheep A and C, respectively) reacted only to the same peptides as sheep F. The observed commonality in peptide epitopes implies that the amino acid sequences recognized, which are conserved among the human and non-human primate AAVs tested, are also conserved in the endogenous ovine AAVs. The data also suggests that these sheep have been exposed to the same or similar viruses.

Several epitopes that were recognized by peptide mapping are located in the unique N-terminus of VP1 and VP1/VP2 overlap which are not ordered in crystal structures determined for the AAV capsids and are also proposed to be buried inside the capsid. In addition, a peptide mapping study for AAV2 by Moskalenko, et al, identified epitopes that were buried in the AAV2 capsid structure, as well as surface associated epitopes [43]. Some of the epitopes identified by Moskalenko, et al, were also identified in this study (Table 1). The AAV capsid in solution is dynamic and undergoes conformational changes upon binding to cellular receptors and uncoating. These changes during the virus life cycle may expose antigenic sites that were previously hidden within the capsid. Zadori, et al [44] have shown that the unique region of VP1 (VP1u), which is internal in the capsid, becomes externalized during the course of infection, possesses phospholipase activity, and is required for infectivity. Recently, specific protein sequence motifs were identified in VP1u that play a role in intracellular trafficking and nuclear localization [45]. Peptides most likely to be antigenic in this region include peptides 26, 323, 450, and 451 (shown in Figure 3). Peptide 26 is an AAV2 peptide that includes the N-terminus of the VP_1/2_ common region. Peptides 450 and 451 are AAV9 peptides that are similar to peptide 34 which is an AAV2 peptide that has previously been shown to contain the epitope for A69 antibody [37]. Peptide 323 (shown in Figure 3) is an AAV8 peptide that includes part of VP2, as well as the N-terminus of VP3. These regions of the capsid are not detectable in the available structures of AAV serotypes solved by X-ray crystallography (Table 1, shaded grey). A similar study using pig sera to evaluate antigenic regions on the capsid of Swine Vesicular Disease Virus (SVDV) also identified novel antigenic regions that were internal or located at subunit interfaces [46]. Common epitopes were recognized for conserved regions of AAV2, AAV5, AAV8, and AAV9 (Table 1, epitope a, b, c, d and e). This cross-reactivity is consistent with reports where immunization with AAV2 capsids led to formation of antibodies that also recognized AAV8 capsids and interfered with subsequent AAV8-mediated gene transfer [47], and with a recently published longitudinal study in pediatric patients with hemophilia [48]. A few peptides listed as positive binders in Table 1 showed some reactivity to secondary antibody alone, although the signal in the control assay was still less than 10,000 LU, these peptides, peptide 44, 354, 355, 485, 486 and 499, represent possible false binders and were omitted from further analysis.

For AAV epitopes, conservation of the sequences recognized and their location is consistent with their reported functional roles in AAV biology. Epitope b, located on the inner surface of the capsid at the inter-pentameric interface, contains amino acids reported to be important to capsid assembly [49]. Peptides 338 and 339 common to AAV2, AAV8, and AAV9, mostly buried or located on the inside surface of the capsid also contain residues shown to be important in capsid assembly [49]. In addition, peptides 368 and 499 contain a residue R432 (in AAV2) involved in genome package [49]. How these regions could become exposed to trigger immunity is currently not clear, but it is possible that presentation of these epitopes could occur once proteolytic digestion of the capsid had occurred to enable peptide loading and presentation to the immune system during the course of natural infection with AAV. Thus for these buried or internally located epitopes, in the case of a natural AAV infection in sheep, antibody neutralizing mechanisms may include inhibition of capsid assembly or genome packaging. In support of a natural AAV infection in sheep, a single report in the literature does describe the isolation of what appears to be AAV-like particles [50], but no further studies were ever conducted to characterize these particles. The presence of what appears to be ovine AAV in nature thus provides a possible explanation for the presence of these cross-reactive antibodies that recognize clinically relevant serotypes of AAV in their sera. Our finding of antibodies against epitopes unique to specific serotypes of AAV suggests that multiple serotypes of ovine AAV with homology to their primate counterparts may exist in nature. Indeed, the successful isolation of multiple serotypes of porcine AAV was recently reported in abstract form [51], making this a promising area for future study. The ability of sheep to serve as a natural host for AAV makes them an attractive model for better understanding and circumventing human immunity to AAV, since such naturally-occurring immunity may well differ from results obtained by immunizing animals with recombinant AAV or AAV capsid-expressing vectors [47]. As a natural host for AAV, and given their closely related immunologic development to humans, it is also possible that sheep may develop memory T cells, enabling them to mount cellular responses to AAV vectors, just as has been seen in human patients. Further studies will be needed to address this important possibility.

### Antigenic regions associated with the capsid surface

As would be expected, the sheep serum also recognized AAV2, AAV5, AAV8, and AAV9 peptides located on the surface of their capsids. Significantly, similar capsid surface antigenic epitopes have been reported for other AAVs. For example, peptide 97 for AAV2 (Figure 4) overlaps with the epitope for AAV2 conformational antibody C37-B reported to block receptor attachment [37]. Mutations in this sequence have also been shown to affect neutralization from human sera or IVIG [52]. For AAV8, peptide 401 (Table 1; Figure 4) is located on a surface variable region, VRVIII, adjacent to the recently mapped ADK8 epitope (residue 586-591) [35]. Binding by this antibody affects infection at a post entry step. The AAV2 and AAV8 surface peptides, along with the surface peptides recognized in AAV5 (Epitope e in Table 1, Figure 4) and AAV9 (peptide 514 in Table 1, colored brown in Figure 4) are located on the protrusions which surround the icosahedral 3-fold axis of the capsid. Residues in this capsid region have been implicated in glycan receptor binding for AAV2, AAV5, and AAV9 [53-56]. Thus it is possible that inhibition of viral entry may contribute to the neutralization observed in the luciferase reporter assays. Similar to the data on the conserved peptides, these surface AAV antigenic epitopes, with sequence and structural differences, are also commonly located on the capsid and likely lead to serotype specific antibody recognition, as observed with the serum from the different sheep.

## Conclusions

The naturally occurring AAV antibodies in the sheep in our study were classified as either low, moderate, or high based on the capsid ELISA. This variation is also observed in serum samples from human subjects. Thus, these sheep could serve as models for evaluating different immune suppression strategies prior to the administration of gene therapy vectors in efforts to optimize strategies to circumvent the immune response. The sheep also represent a useful model for evaluating re-administration of gene therapy vectors, including newly developed AAV vectors whose capsids have been intentionally modified with the goal of generating escape mutants.

To our knowledge, this is the first delineation of the epitopes within the AAV capsid that are responsible for triggering naturally-occurring, functional antibodies in these animals. The presence of these antibodies, coupled with the close parallels between human and sheep physiology, and our re-establishment of sheep with severe hemophilia A, highlight the value of sheep as a large animal model in which to study gene therapy for hemophilia A, and other diseases, in the context of pre-existing humoral immunity to AAV. This will also aid the development of novel strategies for circumventing the immunologic barrier that has thus far thwarted the success of AAV-mediated gene transfer in the clinical arena.
